# Infection control recommendations for radiology departments in Malawi

**DOI:** 10.4102/hsag.v24i0.1035

**Published:** 2019-03-27

**Authors:** Denis Nyirenda, Razana Williams, Wilma ten Ham-Baloyi

**Affiliations:** 1 Department of Radiography, Malawi College of Health Science, Lilongwe, Malawi; 2 Department of Radiography, Nelson Mandela University, Port Elizabeth, South Africa; 3 Faculty of Health Sciences, Nelson Mandela University, Port Elizabeth, South Africa

## Abstract

**Background:**

Guidelines for radiographers contain recommendations related to standard infection control precautions for healthcare-associated infections (HAIs) which are a major cause of mortality and morbidity in hospital settings. However, the implementation of these recommendations has proven to be a challenge in the Malawian radiology departments, as there are no national guidelines or radiology policies for infection control.

**Aim:**

This article outlines the development of infection control recommendations that could facilitate sound knowledge and practices of radiographers regarding infection control.

**Setting:**

Radiology departments in hospitals in Malawi.

**Methods:**

The recommendations were developed based on data from a questionnaire that measured the knowledge and practices of 62 radiographers regarding infection control as well as data from the literature. The Florence Nightingale environmental theory was used as the conceptual framework for the recommendations, while its development was based on steps of the National Institute for Health and Care Excellence. For the format of the draft recommendations, an adapted version of the Appraisal of Guidelines for Research and Evaluation II tool was used.

**Results:**

Issues identified from the responses to the questionnaire and literature resulted in seven sets of recommendations: hand hygiene, personal hygiene, personal protective gear and the use of appropriate equipment, safe handling of sharps and sharp containers, decontamination and cleaning, housekeeping and routine infection control practices.

**Conclusions:**

The recommendations can be further reviewed and implemented to improve the implementation of infection control and to reduce HAIs in resource-constrained settings.

**Keywords:**

healthcare-associated infections; infection control; radiographer; recommendations; Malawi.

## Introduction

Infection control concerns the control of the spread of healthcare-associated infections (HAIs) developed by patients who receive care (Boyle & Strudwick 2010) and it is imperative in maintaining patient safety by reducing the effects of HAIs on the health of patients (Zsuzsanna 2010). Healthcare-associated infections are caused by pathogenic microorganisms, which can be detected in the air, in water and on surfaces (National Health Services Professionals Handbook 2010). The modes of spread of HAIs include direct, indirect and airborne contacts.

The most prevalent HAIs that happen within healthcare institutions include respiratory infections, urinary tract infections, blood infections and wound infections succeeding surgical procedures (Infection Control Certification 2013). As radiographers have direct contact with patients, and other hosts, they are reported as being at a high risk of contracting and spreading infections (Ibeziako & Ibekwe 2006). With the introduction of interventional radiological applications such as opening of intravenous lines, catheterisations performed for gastrointestinal radiological examinations, colonography, cystography and other special imaging modalities, the tendency for accidental and infectious pathogens is on the increase. Such an increase has been associated with the fact that radiological examinations require direct contact between patients and radiographers (Jayasinghe & Weerakoon 2014). Other radiological procedures that result in direct contact between radiographers and patients include urethrography, hysterosalpingography and intravenous urography. An important outcome of the introduction of interventional radiology has been an increase in the number of patients seeking radiological services, which has resulted in an increase in the spread of HAIs. According to Rutala and Weber (2008), the major risk of all such invasive procedures is the introduction of pathogens that can lead to the spread of HAIs.

Standard infection control precautions (SICPs) should therefore be adhered to by radiographers in order to prevent these infections. Radiographers thus require acceptable knowledge levels as well as adequate practices related to infection control in order to prevent the effects of HAIs on patient health and safety. Radiographers’ roles in infection control include maintaining a safe practice environment at all times: selecting appropriate hazard control and risk management and infection control precautions, and application of reduction or elimination techniques in accordance with the health and safety legislation. Additional roles of radiographers include the use of correct principles and applications of disinfectants, methods for sterilisation and decontamination, and precautions recommended for dealing with waste and spillage correctly (Health & Care Professional Council Handbook 2013). Practising infection control in the radiology department, aiming to control and decrease the spread of HAIs, is therefore imperative (Antwi et al. 2015). Radiographers need to understand SICP and should contribute to strategies to decrease the risk of infection to patients, as well as their own personal protection, by complying with these precautions (Sukumar & Yadav 2012). However, low rates of compliance with SICP among healthcare workers, which include radiographers, have been reported globally, as a systematic review found that compliance with SICP is sub-optimal on a global basis (Gammon, Morgan-Samuel & Gould 2008). Standard infection control precautions are paramount to minimise HAIs and high healthcare costs or burdens associated with them (McGaw et al. 2012). Strict adherence to SICP by radiographers may control the risks of HAIs, making it necessary for them to have adequate knowledge and skilled practices about SICP (Phillips & Ker 2006).

### Problem statement

The first author observed that there was low compliance to the application of SICP by radiographers in radiology departments in government referral hospitals in Malawi. For example, radiographers failed to wash their hands after treating each patient and used the same gown as well as disposable gloves for a number of patients (Nyirenda 2017). Furthermore, currently no national guideline, policy or recommendations regarding infection control in Malawi are available for radiographers when implementing infection control in the radiology departments. The observed discrepancies in the application of SICP could be linked to the absence of a national guideline or policy.

### Aim

This study aimed to develop infection control recom-mendations that could facilitate sound knowledge and practices of radiographers regarding infection control in radiology departments in government referral hospitals in Malawi.

### Contribution to the field

The study was unique as it was the first study conducted in this setting and reflects the second phase of a larger study that was conducted to develop an infection control guideline for radiographers in radiology departments in Malawi. The first phase included a questionnaire that was administered to 62 radiographers to determine their knowledge of and practices related to infection control in the respective radiology departments (Nyirenda 2017).

### Definition of key concepts

#### Healthcare-associated infections

Healthcare-associated infections refer to infections associated with healthcare delivery in a hospital or ambulatory setting (Friedman et al. 2011). In this study, HAIs are infections one gets upon exposure to infected places where radiological examinations are performed.

#### Infection control

Infection control refers to processes and activities aimed at identifying and reducing the risks of obtaining and transferring prevalent or widespread infections among people (David & Famurewa 2010). For the purpose of this study, infection control refers to a practice of ensuring that the environment is free from pathogenic microorganisms.

#### Radiographer

A radiographer is an allied health professional, who is registered with an appropriate professional board, and performs diagnostic examinations on patients using a variety of imaging modalities, including radiography, computed tomography (CT), magnetic resonance imaging (MRI), mammography and cardiovascular interventional technology (Ehrlich & Daly 2009). In this study, a radiographer is a person who performs radiological diagnostic examinations and is involved in infection control. Radiographers are the target population for the infection control guideline developed in this study.

#### Recommendations

Recommendations are suggestions regarding the best course of action, which are intended to assist providers and recipients of healthcare, and other stakeholders, to make informed decisions (WHO 2012). Recommendations that are developed in this study include written statements expressing procedures or protocols on how implementation of infection control should be conducted in a radiology department.

## Research method and design

### Setting

During the period of the study, there were 31 radiology departments in Malawi of which 27 were in government district hospitals and four in government referral hospitals. The recommendations were developed for radiology departments in four government referral hospitals located in the major cities of Malawi. The four government referral hospitals were targeted because those are the only hospitals in Malawi that conduct invasive procedures and, therefore, carry a bigger risk for HAIs.

### Procedure

The recommendations were developed by the first author, while being supervised by the second and third authors in April 2017 based on data from a questionnaire that measured the knowledge and practices of 62 radiographers regarding infection control as well as a narrative literature review. The narrative literature review used the following databases: EBSCOhost including CINAHL with Full Text, Health Source-Consumer Edition, Health Source: Nursing/Academic Edition, MasterFILE Premier, MEDLINE, PsycINFO. In addition, ScienceDirect and Google Scholar were searched and a manual search in Google and the reference lists of relevant articles was also done. Keywords included “infection control” OR “infect*” AND “radiograp*” AND “principle*” OR “practice*” OR “measure*”.

The conceptual framework for the recommendations entailed Florence Nightingale’s environmental theory (Nightingale 1969). This theory encourages the configurations of environmental settings appropriate for restoration of health (Wayne 2014). It was used as no published radiography model or theory related to infection control and the environment was available. Nightingale emphasised the responsibility of nurses to put patients in the best situation for the environment to allow them to heal (Nightingale 1969). The patient (client) and a radiographer (instead of a nurse) and the major environmental concepts must be in balance. In the case of this study, a radiographer’s responsibility was to maintain an environment that will prevent patients from obtaining HAIs during a radiological procedure (George 2011).

For this study, two major aspects of Florence Nightingale’s environmental theory were identified, which need to be controlled for the prevention of HAIs: health of houses and cleanliness, the latter being subdivided into cleanliness of the rooms and personal cleanliness (see Table 1).

**TABLE 1:** Application of the major areas of Nightingale’s environmental theory.

Major areas (Nightingale 1969)	Description of the study (Nyirenda 2017)
Health of houses	A clean environment and waste disposal, for example disposal of needles after use, how to take care of the sharp containers before disposal to prevent HAIs, etc.
Cleanliness	
Cleanliness of the rooms	Cleaning, disinfecting, damp dusting and checking radiographic equipment for cleanliness prior to using them to prevent HAIs.
Personal cleanliness	Use of personal protective gear and the use of appropriate equipment (e.g. gloves, mask and goggles), as well as personal body cleanliness (hand washing, hand disinfecting agents, showering/bathing/washing, brushing teeth, covering of cuts or wounds, cutting long fingernails, removal of jewellery during working hours as well as providing each patient with a clean gown to prevent healthcare-associated infections).

Furthermore, the recommendations were developed based on steps derived from the National Institute for Health and Care Excellence (NICE) method (NICE 2012), and for the format of the draft, an adapted version of the Appraisal of Guidelines for Research and Evaluation II (AGREE II) tool (AGREE 2009) was used (see Table 2).

**TABLE 2:** Steps in the development of the draft recommendations.

Steps	Activity and/or application
Step 1: consider recommendations’ remit	Identification of the scope and/or objectives and purpose of the recommendations.
Step 2: identify key issues to be included in the recommendations	The draft recommendations were developed based on gaps identified in data derived from a questionnaire exploring and describing the knowledge and practices of 62 radiographers regarding infection control (referring to the issues of houses, cleanliness of rooms and personal cleanliness, as outlined in Table 1) in radiology departments of four referral hospitals in Malawi (Nyirenda 2017).
Step 3: undertake scoping literature search	A critical literature review was used to substantiate the findings from the questionnaire (see step 2).
Step 4: start drafting the plan and prepare the first draft	Drafting of the recommendations was done using the data derived from the questionnaire and the literature review used. For the format of the draft recommendations, an adapted version of the AGREE II tool for guideline development was used (AGREE 2009).
Step 5: hold stakeholder workshop	The recommendations were reviewed by the second and third authors who are experienced in developing recommendations in the fields of radiography and nursing.
Step 6: consult on the draft scope	A meeting for consensus between the authors was held.
Step 7: finalise the scope after consultation	Relevant suggestions made by the second and third authors were considered.

*Source*: Adapted from National Institute for Health and Care Excellence (NICE), 2012, *The guidelines manual. Process*, viewed 02 March 2017, from http://www.nice.org.uk/process/pmg6

AGREE II, Appraisal of Guidelines for Research and Evaluation II.

## Quality of data

In this study, face validity of the recommendations was ensured by requesting the review of the recommendations by the second and third authors, while reliability of the recommendations was ensured by applying NICE’s validated steps (NICE 2012) during its development. The conceptual framework for the recommendations entailed the environmental theory of Florence Nightingale (Nightingale 1969). The content of the recommendations was based on data from the self-administered questionnaire and a narrative literature review. The adapted version of the AGREE II tool (AGREE 2009) was used for the format of the draft recommendations.

## Results

The title of the recommendations reads ‘Recommendations to facilitate infection control for radiology departments in Malawi’. The recommendations aim to facilitate sound knowledge and practices of radiographers regarding infection control among radiographers in radiology departments in Malawi. Seven sets of recommendations were developed (see Table 3).

**TABLE 3:** Sets of recommendations.

Set of recommendation†	Objectives‡	Recommendations
1. Hand hygiene (personal cleanliness)	To mechanically remove soil and debris from the skin and reduce the number of transient microorganisms.	Hand hygiene (World Health Organization 2012): Before touching a patient. Before clean and aseptic procedure. After body fluid exposure risk. After touching a patient and at the end of a shift. Alcohol-based hand rub technique (Harrogate and District NHS Foundation Trust 2016): Ensure you are ‘bare below the elbows’. Apply enough antiseptic hand rub (about a teaspoonful) to cover the entire surface of hands and fingers. Ensure the solution covers the wrist surfaces. Rub the solution vigorously into hands, especially between fingers and under nails until the solution gets dried (about 20–30 s).
2. Personal hygiene (personal cleanliness)	To reduce the number of microorganisms that may be present in their bodies and those that may harbour in their jewellery.	Rules of personal hygiene (Lister & Inamdar 2013; Netherlands Development Organisation 2014): Shower, bath or wash thoroughly every day. Keep hair and nails short, clean and neat. Cover open cuts and wounds. Brush your teeth at least twice daily and use a breath refresher as needed throughout your shift. Always wear clean clothes. Always remove jewellery during a radiological procedure.
3. Personal protective gear and the use of appropriate equipment (personal cleanliness)	To protect themselves, fellow healthcare workers and patients from microorganisms from contaminating hands, eyes, clothing, hair and shoes.	Inspection, cleaning and maintenance of personal protective equipment (PPE) (Australia Workforce Health 2015): Inspect the PPE for signs of damage prior to use. Clean/decontaminate all reusable PPE. Avoid using cleaning agents that are likely to scratch surfaces of PPE. Store PPE in clean, sealed containers such as plastic tubs with lids. Ensure that the PPE is kept clean in between usages. Remove damaged PPE from use.
4. Safe handling of sharps and sharp containers (health of houses)	To avoid occupational exposure to microorganisms that may be found in the blood and other body fluids which may occur because of needle stick (sharp) injuries.	Safe use of sharps (Loveday et al. 2014): Do not pass sharps directly from hand to hand. Keep the handling of sharps to a minimum. Do not recap bent or disassemble used needles, managing sharps and sharp containers (Kenya Ministry of Public Health and Sanitation & Ministry of Medical Services 2010): Position the sharp end of instruments away from self and others. Dispose of used sharps immediately in designated puncture and leak-proof containers labelled with a biohazard symbol. If injured by sharps, contact your supervisor immediately. Put sharps containers as close to the point of use as possible and practical, at a convenient height and ideally within arm’s length. Label sharps containers clearly with a biohazard symbol so that people will not unknowingly use them as garbage or trash containers. Keep sharps containers in the area where sharps are being used. Do not place sharp containers where people might accidentally put their hands on them. Do not fill sharps containers above the three-quarters full mark. Seal the container when it is three-quarters full and do not reopen it. Never reopen, empty or reuse a sharps container after closing and sealing it.
5. Decontamination and cleaning (cleanliness of rooms)	To prevent potentially harmful microorganisms reaching a susceptible host in sufficient numbers to cause infection.	Decontamination (Kenya Ministry of Public Health and Sanitation & Ministry of Medical Services 2010): Decontaminate large surfaces or instruments that might have come into contact with blood and body fluids. Wipe them with a cloth soaked in the chlorine solution. Immediately after use, place all instruments in an approved disinfectant such as chlorine solution for 10 min. Remove instruments from chlorine solution after 10 min and immediately rinse them with cool water to remove residual chlorine before thoroughly cleaning them. Once instruments and other items have been decontaminated, cleaned and sterilised or high-level disinfected. Dry the instruments thoroughly.
6. Housekeeping (cleanliness of rooms)	To reduce the number of microorganisms that may come into contact with staff, patients, visitors and the community in order to provide a clean and pleasant atmosphere for patients and staff.	Dusting (Kenya Ministry of Public Health and Sanitation & Ministry of Medical Services 2010): Wet cleaning cloths or mops with cleaning solution contained in a basin or bucket. Avoid dry dusting; dust cloths and mops should never be shaken to avoid the spread of microorganisms. Avoid raising dust. Perform dusting in a systematic way, using a starting point as a reference to ensure that all surfaces are reached. Chairs, lamps, tables, table tops, chest stands, hand rails and counters (Kenya Ministry of Public Health and Sanitation & Ministry of Medical Services 2010): Wipe daily and whenever visible soiled with a damp cloth, containing disinfectant cleaning solution. Use a disinfectant when contamination is present (e.g. blood or other body fluid spills).
7. Routine infection control practices (cleanliness of rooms)	To minimise the risk for transmission of infection among patients and personnel in the radiology departments.	Routine infection control practices (Ehrlich & Coakes 2017; Tugwell & Maddison 2011; UNC Health Care 2016): Clean all radiologic equipment on a routine basis (e.g. weekly), whether soiled or not soiled. All radiographic accessories labelled for single use should not be reused. Clean or decontaminate reusable radiographic accessories to remove all visible matter/debris prior to placing accessories in the central processing and distribution container in readiness for sterilisation. Between each patient that has undergone examination, thoroughly clean X-ray tables, vertical stands and any other items that come into contact with patients. Clean and disinfect portable radiographic equipment before and after entering the room of a patient or ward. Dust daily the overhead tube, spot film devices, image intensifiers and television monitors. Dust weekly the overhead tracks for ceiling-mounted equipment using a vacuum cleaner. Wash weekly control stands and spot film devices with disinfectant. Clean lead rubber gloves and aprons weekly using disinfectant, dilute bleach or a germicide cloth. Clean, wash and disinfect anatomical markers with gel weekly and use only once per patient. Cover all portable X-ray cassettes and grids with a disposal, clear plastic cassette cover to prevent contamination. Clean all anatomical markers, positioning pads and sponge pads with an approved disinfectant after each patient/radiographic procedure. Always provide a patient with clean attire (e.g. a clean hospital gown) for all radiological examinations that require the use of a gown.

† , applicable to major aspects of Florence Nightingale’s environmental theory.

‡ , to ensure that radiographers have adequate knowledge and practices.

## Discussion

Globally, infection control in radiography departments is recognised as imperative and this is visible through various infection control policies. These policies focus on a range of infection control principles, including hand hygiene (e.g. handwashing and alcohol-based hand rub), isolation (contact, airborne and droplet), the use of personal protective equipment, as well as managing contaminated equipment and cleaning and disinfecting of equipment (Department of Radiology 2009; Montana State Hospital 2017; Radiology Compliance Branch 2015). The developed recommendations focused on hand and personal hygiene, personal protective gear and the use of appropriate equipment, safe handling of sharps and sharp containers as well as housekeeping and cleaning and routine infection control practices. These practices have been found to be effective in reducing HAIs if well adhered to by radiographers (Chingarande & Chidakwa 2014; Crofton & Foley 2018). In order to adhere to the infection control practice, implementation of the recommendations is therefore important, which was not done in this study. It is thus suggested that the recommendations should be reviewed by experts prior to their implementation (NICE 2012). The expert panel can include a Ministry of Health official representing radiographers, principal radiographers from government referral hospitals and radiography lecturers from radiography training institutions in Malawi. Recommendations for clinical infection control provide comprehensive endorsements for preventing HAIs in hospitals (Loveday et al. 2014). Similarly, the recommendations for infection control developed by the researcher can then be used to facilitate the sound infection control knowledge and practices of radiographers in radiology departments in hospitals in Malawi. However, for proper implementation of the recommendations, leaders (management and radiographers) at all levels of the hospital should be committed to support the facilitation of its implementation (Registered Nurses’ Association of Ontario 2012). Currently, the radiology departments have inadequate infection control resources, which include patient gowns, personal protective equipment (e.g. gloves) and cleaning materials (e.g. detergents). A context analysis should therefore be performed to identify the required resources for implementation of the recommendations. The recommendations should also be made available in various formats (print and electronic) to the radiographers in order to enforce their implementation. Furthermore, documentation (e.g. logs or charts) indicating the infection control practices and procedures that need to be followed should be made available and radiographers should be trained to complete these forms (Radiology Compliance Branch 2015).

Furthermore, there is a need to further explore the knowledge and practices of radiographers regarding infection control in Malawi. Based on this, the recommendations can be further developed and reviewed. An intervention study using a pre- and post-knowledge and practice questionnaire as well as workshops on the sets of recommendations, in order to evaluate the recommendations’ effect on the infection control knowledge and practices of radiographers, can be conducted. The recommendations, once reviewed and further developed, should be updated with recent literature at least every 5 years so that the recommendations are current.

### Limitations of the study

The study has some limitations. The recommendations were developed by a single author and have not been reviewed by experts in the field. It is suggested to have the draft recommendations reviewed by experts before implementation (NICE 2012). In order to develop the recommendations, only the databases of the university to which the researcher had access were used, which may have resulted in some documents not being available, and therefore not being included. The recommendations have not been implemented and suggestions for a pilot study to implement the recommendations were made. The current recommendations may not be applicable for all radiographers in Malawi because the study was limited to radiology departments in the four government referral hospitals. Suggestions to replicate the study using a bigger sample size were made.

## Conclusion

Infection control recommendations including seven sets of recommendations were developed for radiographers who are operating in radiology departments of Malawian government referral hospitals. The recommendations identified ways to further facilitate infection control. The recommendations require expert review for further validation. After implementation, the seven sets of recommendations may provide radiographers with the knowledge of infection control as well as guidance on sound infection control practices in radiology departments. However, for successful implementation of the set of recommendations, the radiographers will also need resources such as gowns, gloves and detergents, as well as support from the management or authorities. Engagement of professional associations in the implementation of the set of recommendations will be crucial as they are the key in safeguarding the scope of practice.
